# Political Philosophies and Positive Political Psychology: Inter-Disciplinary Framework for the Common Good

**DOI:** 10.3389/fpsyg.2021.727818

**Published:** 2021-12-13

**Authors:** Masaya Kobayashi

**Affiliations:** Graduate School of Social Sciences, Chiba University, Chiba, Japan

**Keywords:** citizenship, justice, well-being, political philosophy, positive psychology, eudaimonia

## Abstract

This manuscript explores the relationship between positive psychology and political philosophy, revealing an inter-disciplinary approach that speaks to the concerns of the common good. Since positive psychology has been expanding its reach into social and political spheres, its relationship to philosophical arguments has been worthy of exploration. Positive psychology is associated with utilitarianism, and aspects of hedonic psychology. However, an alternative concept of eudaimonic well-being has enabled this psychology to have links to other political philosophies. Therefore, this manuscript provides an overview of contemporary political philosophies: first, it discusses the debate between liberalism and communitarianism, and secondly, it summarizes the subsequent developments of liberal perfectionism, capability approach, and deliberative democracy. Then, the configuration of these political philosophies is indicated by the figure of two axes of “individual/collective” and “ethical/non-ethical.” The following section compiles the inter-relationships between the conceptions of citizenship, justice, and well-being, regarding the main political philosophies: egoism, utilitarianism, libertarianism, liberalism, communitarianism, and conservatism. Utilitarianism is associated with happiness, while liberalism and libertarianism rely on the concept of rights, which is almost equal to the idea of justice. Accordingly, utilitarianism is a philosophy of well-being, while liberalism and libertarianism are philosophies of justice. However, there is little connection between well-being and justice in these philosophies because the two kinds of philosophies are incompatible. The latter kind criticizes the former because the maximization of happiness can infringe on people’s rights. Moreover, these philosophies do not particularly value citizenship. In contrast, communitarianism is intrinsically the political philosophy of citizenship most attuned to increasing well-being, and it can connect an idea of justice with well-being. The final part offers a framework to develop an inter-disciplinary collaboration. Positive psychology can provide the empirical basis of the two axes above concerning political philosophies. On the other hand, the correspondence makes the character of political philosophies clearer. While libertarianism and liberalism correspond to psychology as usual, utilitarianism and communitarianism correspond to positive psychology, and the latter can be regarded as positive political philosophies. This recognition leads to the interdisciplinary framework, enabling multi-disciplinary collaboration, including work with the social sciences, which could benefit the common good.

## Introduction: Psychology and Political Philosophy

### Positive Psychology and Utilitarian Tradition

This manuscript will explore “Psychology for the Common Good” by examining the association between psychology and philosophy, more concretely, positive psychology in the context of political philosophies. Positive psychology investigates the good life scientifically. On the other hand, philosophy examines ideal ways of living and suggests ways to improve our society. Combining philosophical inquiries with contemporary science can assist us in exploring new ideas and practices related to personal and public well-being. This article seeks to accomplish this task by extending the recent developments of positive psychology.

Let us begin by reviewing the basics. There are several major contemporary political philosophies: utilitarianism, libertarianism, liberalism, communitarianism, or republicanism. Although there are various sub-types, intermediaries, and combinations, this manuscript first focuses on these major representative philosophies for making the relationships between political philosophies and psychology clear.^1^

Positive psychology has been frequently associated with utilitarianism within these political philosophies (Veehhoven, 2003; Tännsjö, 2007). The reason for this is that this philosophy typically argues for the maximization of happiness for all people concerned. The classical formulation is Jeremy Bentham’s “the greatest happiness of the greatest number.” Correspondingly, positive psychology often utilizes the indicators of “subjective well-being” explored by Ed Diener, and the form of psychology that measures well-being in this way is sometimes called “hedonic psychology” (Kahneman et al., 1999).

However, there has emerged a considerable amount of criticism against this ascription. Some scholars have argued against using pleasure as a measurement of well-being, and they have objected to such ideas as Hedonia, putting forth instead a new concept and measurement based on the word “Eudaimonia,” which originated in ancient Greece. They pushed forward the idea of “eudaimonic well-being,” suggesting that conceptions such as growth, self-realization, engagement, and meaning constitute eudaimonic well-being.

This was followed by a heated debate between those arguing for subjective well-being (e.g., Kashdan et al., 2008) and those supporting eudaimonic well-being (Waterman, 1993, 2008, 2013). This debate seems to empirically demonstrate the conclusion based on the following: the two indicators are correlated but independent, and eudaimonic well-being has a higher correlation with eudaimonic functioning such as self-realization, endeavor, meaning, elevation, relation with others, and creativity, while subjective well-being correlates more highly with hedonic pleasure or enjoyment.

Therefore, it follows from this debate that the philosophical underpinnings of positive psychology should not be confined to classical utilitarianism. While the classical utilitarianism of Bentham is viewed as hedonic and quantitative, J. S. Mill later proposed qualitative utilitarianism. Moreover, there have appeared various variations of consequentialism or welfarism from this tradition in contemporary philosophy. Consequentialism signifies that specific normative properties depend on consequences, which are calculated by the sum of pleasure in classical utilitarianism but are inferred by more sophisticated ways in non-utilitarian consequentialism today.

Welfarism in economics is a kind of consequentialism, which regards the impact on welfare as morally significant. This economic idea depends on the conception of utility, and it assumes that social welfare can be conceived as an aggregation of individual utilities. As utility means the degree of pleasure or satisfaction an individual receives from economic activity, it is more or less a hedonic conception. In contrast, welfarism in a broad sense, signifies “nothing but welfare matters, basically or ultimately, for ethics” (Sumner, 1996, p.184), and it is neither necessarily consequential nor aggregational.

As qualitative utilitarianism recognizes the difference in the quality of pleasure, it is closer to communitarianism discussed below than original utilitarianism. In contrast, non-utilitarian consequentialism is frequently unrelated to a specific human description. Accordingly, there is little relationship between these currents originating from utilitarianism and psychology. In addition, while economic welfarism is basically hedonic and preserves the utilitarian element, welfarism in general is compatible not only with utilitarianism but also with the other political philosophies discussed below (Sumner, 1996, p. 186).

It would then be necessary to examine the relationship between the other political philosophies and positive psychology.

### Criticism Against Positive Psychology and Its Two Frontiers

Apart from this debate, positive psychology has been criticized on various points since its birth (Lazarus, 2003). For example, existential psychologist Paul Wong pointed to its problems or limitations: elitism, scientism, positive-only focus, componential rather than holistic thinking, value-neutral position, lack of comprehensive theory, positivist paradigm, dependence on “quick-and-dirty” measures, cultural critiques (Wong and Roy, 2018). Among such weak points, two “challenges to positive psychology” (Gable and Haidt, 2005, p. 107) are especially prominent: first, focus on the positive side disregarding the negative side; secondly, little progress in research on positive institutions and communities.

The second point is closely related to the subjects of political philosophy. One of the most scathing criticisms against positive psychology is that it is permeated with a Western-centered or American-inspired brand of individualism. As a result, it does not sufficiently deal with a societal, cultural, and political force (Becker and Marecek, 2008).

In reality, the manifest of positive psychology enumerated its three pillars as “subjective emotion, individual traits, and institution”(Seligman and Csikszentmihalyi, 2000). Accordingly, the institutional dimension has been theoretically considered one of the core subjects of positive psychology. The monumental article explained the third element in the following manner: “At the group level, it is about the civic virtues and the institutions that move individuals toward better citizenship: responsibility, nurturance, altruism, civility, moderation, tolerance, and work ethics” (Seligman and Csikszentmihalyi, 2000, p. 5). Thus, this element precisely signifies just the civic virtues and institutions for citizenship.

Nonetheless, it cannot be denied that its focuses have been on the former two, namely, the investigations of personal well-being and character strengths. As a result, early hopes for exploring new fields like positive anthropology and positive social sciences have gone “unfulfilled” (Gable and Haidt, 2005, p. 108).

Accordingly, as a response to this criticism, there have been some noteworthy attempts at deploying positive psychology for the development in social or political spheres (Haidt, 2012; Kern et al., 2019; Ward et al., 2021) or the application in policy evaluation and policy studies (Diener and Seligman, 2004; Diener et al., 2009). The conception of positive social science or positive organizational studies has already been put forth (Cameron et al., 2003). Nevertheless, it would still be essential to examine further the relation to social or political studies.

Moreover, the first point has relevance also in the collective spheres. Unfortunately, the world remains full of negative political and social phenomena such as misery, poverty, conflicts, war, corruption, dictatorship, and pandemic like COVID-19. Thus, there are many issues of “political philosophy (and political sciences) as usual”. In contrast, positive ideas, including justice, fairness, and the common good, are included within central political philosophy conceptions. Accordingly, it is necessary to deal with both dark and bright sides in social or political spheres.

Fortunately, there have appeared new waves of positive psychology for amending the two weaknesses. Second wave positive psychology aims to integrate the positive and negative sides dialectically, exploring the complex relationships between both sides (Wong, 2011; Ivtzan et al., 2016). Then, third wave positive psychology proposes to go beyond the inquiry of individuals for that of groups and systems with the greater complexity, utilizing more interdisciplinary, multicultural, and various methodologies (Lomas et al., 2020).

**FIGURE 1:**
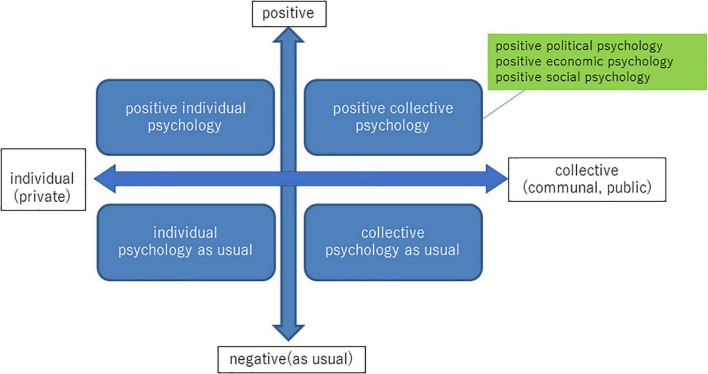
Positive individual/collective psychology.

Figure 1 illustrates such a development. The vertical axis is ‘‘positive/negative (or as usual)’’ as ‘‘positive psychology/psychology as usual.’’ The horizontal axis is ‘‘individual (or private)/collective (or communal, public).’’ Thus, the positive collective psychology in the first quadrant consists of positive psychologies in public spheres, including politics, economy, and society. So then, the research on political themes and well-being can be called ‘‘positive political psychology^2^ similar to “positive social psychology” (Lomas, 2015) concerning sociocultural well-being in general.

The themes of justice and citizenship are primarily related to the political sphere, and this manuscript investigates them from the angle of positive political psychology.

## Major Political Philosophies and Recent Developments

### The Debate Between Liberalism and Communitarianism: Justice and the Good Life

The most salient contemporary philosophical alternatives to utilitarianism are liberalism and libertarianism. However, in general, these are not related to psychology. These political philosophies assume that there are many conceptions of the good life grounded in the value-system or worldview of today, and it could repress the other to base justice on one of them. Thus, the argument is that it would be impossible to take one of them as the basis for public decisions and policies, and the only way of agreeing on justice beyond such different views would be to rely upon the concept of rights. Accordingly, these theories are called deontology or rights-based theories.

There are intense controversies over welfare issues between egalitarian liberalism and market-oriented libertarianism: the former can provide a philosophical foundation of the welfare state by its conception of welfare or social rights, while the latter argues for small states with little welfare emphasizing the property rights. Nevertheless, both share a deontological theoretical construction based on the conception of rights.

These theories value modern ideals such as autonomy, equality, individuality. They rely on the idea that each human being is crucial in oneself, and an individual’s choice by their free will needs to be respected, whether the choice seems good or bad from some outside ethical perspective. Thus, these are individualistic and non-ethical.

The most well-known contemporary theory among these is John Rawls’ liberalism described in *A Theory of Justice*. The cardinal idea of liberalism is summarized as “the priority of justice over the good” (Rawls, 1971). Pursuing the good life is not prohibited in private lives, but it is not related to the public sphere. Instead, justice in public decisions should be grounded upon the concept of rights, bracketing the difference over the conception of the good life. Although there are variations of theoretical notions, most representative liberalists and libertarians supported the idea of state neutrality among various conceptions of the good life and its virtues, and this thesis came to be the central conception of mainstream liberalists (Nozick, 1974; Dworkin, 1978; Ackerman, 1980; Larmore, 1987; Kymlicka, 1989; Nagel, 1991).

As most of these thinkers support the deontological construction based on the conception of rights, these will be termed deontological rights-based liberalism. This word here signifies moral theories requiring people to accomplish what people ought to do (deontic theories) on the reasoning of the priority of the right over the good in opposition to virtue theories and consequentialism.

“The priority of justice over the good” in these thoughts was challenged by Michael Sandel (1982). According to him, it is impossible to make public decisions based only on the concept of rights. For instance, the conceptions of rights in liberalism and libertarianism are critically opposed to each other. On the one hand, Rawlsian egalitarian liberalism argues for distributive justice and a welfare state. On the other hand, libertarianism attaches importance to property rights and denies the right to welfare. As a result, it is almost equal to neo-liberalism in economics in the negation of the welfare state and calling for a small state.

From the outside perspective, the difference originates from their views on the good associated with various worldviews. It is difficult to decide what justice is regarding the environment, security, bioethics, and welfare, without mentioning values. Consequently, it is necessary to conduct public discussions on these issues concerning the good life and determine what is just through public deliberation. That is to say, justice is related to the conception of the good life. In other words, the right is concerned with the good. This idea is the core of the communitarian concept of justice. Therefore, it is important to dare discuss these public issues referring to the good life to revitalize democratic politics.

This argument leads to the debate between liberalism and communitarianism (Mulhall and Swift, 1996). Sandel’s criticism against Rawls’ *A Theory of Justice* includes the view of self (Sandel, 1982). Rawls assumed that people in the original position knew nothing about their concrete situations such as age, sex, talent, status, and income under the “veil of ignorance” when they considered and agreed with the principles of justice in the hypothetical social contract. Sandel called this conception of self the “unencumbered self” and pointed out that the actual self is situated in various contexts and constituted by the ethical ideals of the good life in such contexts as the family and multiple communities.

Similarly, Alasdair MacIntyre criticized modern ethics and revived virtue ethics (MacIntyre, 1981). The sociologist Amitai Etzioni emphasizes the importance of responsibility as well as the concept of rights and promotes the responsive communitarian movement (Etzioni, 1993).

In addition, such an ethical orientation is frequently considered to be important in the political sphere. For example, Sandel typically argued for the resurgence of republicanism as a public philosophy in America instead of the liberalism that has been dominant since WWII (Sandel, 1996). Republicanism originates in *res publica* in ancient Greek and Rome, and it means active political participation for self-government by people with civic virtue. If people lack civic virtue, they tend to fall into political apathy or become manipulated by demagogues. Thus, civic virtue has a vital role in making democracy sound and better in quality.

Although liberalism sometimes supports republicanism, it respects the institutional mechanism against dictatorship, typically separation of powers. Accordingly, it sometimes supports people’s political participation: this version is liberal republicanism (Ackerman, 1993/2000). Nevertheless, liberalism, including even this version, tends to disregard the ethical aspect of republicanism. In contrast, communitarianism emphasizes the vital significance of civic virtue for political participation. It advocates civic virtue as one of the essential human virtues, and therefore it frequently accompanies republicanism to be termed communitarian republicanism.

In sum, while liberalism and libertarianism are individualist and non-ethical, especially concerning public spheres, communitarianism has an ethical and communal (or public) orientation: it attaches importance to various collaborative activities and communities, as well as to the good life sustained by morality and virtue, not only in private lives but also in public lives. As this debate presents one of the essential issues in contemporary political philosophy, the two ethical and communal (or public) axes found in this debate will be helpful to overview the other recent developments in the next section.

### Beyond Deontological Rights-Based Liberalism

#### Liberal Perfectionism

Partly due to the impact of the communitarian charge, some new approaches from within liberal currents are somehow opposed to typical deontological rights-based liberalism, and they have led to the “troubled dominance of the liberal paradigm” (Christiano and Christman, 2009, p. 5).

First, concerning the ethical dimension, the neutrality thesis was amended even within liberal theorists. Some admitted that any liberal belief of state neutrality could not be consistently justified: some ethical goals and ideals may be supported by the state because many virtues or conceptions of the good such as love and friendship are entirely uncontroversial. In sum, the thesis is an illusory myth (Beckman, 2001, pp. 262–264).

Accordingly, there appeared discussions between state neutrality principle and perfectionism, assuming that the state should favor some valuable conceptions of the good (Wall and Klosoko, 2003, pp.13–16; Merrill and Weinstock, 2014). For instance, some critics pointed to value commitments in the proponents of state neutrality for moral equality, liberty, and democracy (Haksar, 1979; Macedo, 1990). Related discussions illuminated that there are various versions of neutrality principles concerning the scope, formulation, and stringency.

For example, Joseph Raz argued that the achievement of strict political neutrality is almost impossible and proposed liberal perfectionism based on moral pluralism, regarding autonomy as ethics of well-being. According to him, states have the duty to provide conditions for facilitating or defending objective well-being: much perfectionist political action need neither be coercive nor controversial. This is not necessarily grounded in a unitary comprehensive conception of the good life (Raz, 1986).

Thus, several theorists following Raz have insisted on liberal perfectionism (Hurka, 1993; Sher, 1997; Wall, 1998). Most of these are the weak thesis of perfectionism trying to balance with non-perfectionist regard: perfectionism can defend individual freedom and limited government, frequently based on value pluralism and the ideal of autonomy as perfectionist good (Wall and Klosoko, 2003, pp. 17).

Although this liberal perfectionism can contain deontological elements such as the state’s duty in Raz’s conception, the duty originates from the good rather than the right. Therefore, this is opposed to deontological rights-based liberalism due to rejecting “the priority of the right over the good.”

#### Capability Approach: Consequential, Perfectionist, and Political Liberalism

Secondly, a noteworthy attempt from within economics, which is also concern with the ethical dimension, appeared. As is well-known, Amartya Sen intrinsically criticized the new welfare economics within the tradition of utilitarianism. He regarded utilitarianism as a combination of three requirements: welfarism, sum ranking, and consequentialism. Sum-ranking had already been criticized within economics, and there remained only the (non-utilitarian) consequentialism after his criticism of welfarism (Sen, 1979, 1987, p.39).

While mainstream economics is based on the individualistic construction associated with egoism, Sen criticized the self-interest maximization view of rationality behind “economic man” in neo-classical economics (Sen, 1977). He paid attention to the reality of interdependence, departing from the shared assumption of both utilitarianism and deontology: individuals are independent and separate. He furthermore focuses on “sympathy,” deriving from A. Smith, and “commitment,” an attitude to pursue a value without self-interest. Smith, a founding father of modern economics, was also a moral philosopher: his two masterpieces are *The Theory of Moral Sentiments* (1759) and *the Wealth of Nations* (1776), corresponding to moral philosophy and economics. Thus, Sen tries to recover the bridge between the ethical and engineering approaches in economics, each of which existed in the origin of modern economics (Sen, 1987).

Then, he proposed the concept of functioning (achievement of a person) instead of utilitarian welfarism and defined the concept of capability as an “alternative combination of functioning the person can achieve, and from which he or she can choose one collection” (Sen, 1993, p. 31; Sen, 1999, p. 9).

On the other hand, he valued the concepts of rights for their essential role in overcoming the shortcoming of welfarism. Accordingly, he tried to integrate consequentialism and deontology by proposing the concepts of a “coherent goal-rights system,” emphasizing the necessity of freedom (Sen, 1987). Thus, his approach is close to liberalism in his focus on freedom in terms of capability. Instead of Rawl’s contractual reasoning, he proposed an impartial and objective approach of justice based on the capability approach (Sen, 2009). Accordingly, this approach is regarded as a (non-utilitarian) consequential (non-deontological) liberalism.

In addition, philosopher Martha Nussbaum collaborated with Sen in the quality of life project at the World Development Bank because Sen invited her to cooperate. This project influenced the idea of human development embodied in the Human Development Index of the United Nations Development Program.

At the time, Nussbaum proposed a kind of Aristotelian philosophy (internal-essentialism). Although Sen does not support constructing a universal and comprehensive list of capabilities (Sen, 1993), Nussbaum presented the list of “thick vague theory of good” (Nussbaum, 1987, 1990, 1992, 1993). Therefore, her theory was characterized as a “liberal perfectionist egalitarian approach” (Arneson, 2000): perfectionistic because of her Aristotelean objective theory of the human good and liberal because of the conception of capability.

However, in 1998, Nussbaum surprisingly radically shifted her approach to replace the conception of human capabilities from the Aristotelian framework into Rawlsian political liberalism (Rawls, 1993; Nussbaum, 1998, 2000; Deneulin, 2002). Consequently, Nussbaum moved from the Aristotelian liberal perfectionist capability approach to the Rawlsian liberal capability approach, refuting Raz’s perfectionist liberalism (Nussbaum, 2011). Thus, while Sen reached consequential liberalism as an alternative to Rawlsian deontological liberalism, Nussbaum turned to Rawlsian political liberalism.

#### Deliberative Democracy: Liberal/Critical vs. Republican Version

Thirdly, theories of deliberative democracy have surged since 1990’ with regard to the communal or public dimension. Before then, the predominant theories of liberal democracy theories do not value civic participation and discussion, for example, in the elitist theory of democracy (J. Schumpeter) and political theories grounding on the assumption of self-interests (such as pluralism and rational choice theory). Instead, deliberative democracy has focused on citizens’ democratic reflection and debate and regards deliberation as central to decision-making (Bohman and Rehg, 1997, p. ix) for the common good or public good to increase the quality of democracy.

The deliberative process can change people’s preferences before decision-making. This transformative nature of deliberation is different from bargaining and aggregation of preferences. Therefore, in contrast to aggregative democracy, deliberative democracy requires citizens to transcend their private self-interests predominant in the market and search for public interests. For this purpose, public forums for deliberation and reason are evaluated and proposed, exemplified by empirical research and proposals such as deliberative polls and deliberative day (Fishkin, 1991, 1995; Fishkin and Laslett, 2003; Ackerman and Fishkin, 2004).

As the similarity to republicanism is evident, republicanism can be a type of deliberative democracy (cf. Forst, 2001). Some try to bridge these two (Pettit, 1997; Peterson, 2009; Hurt, 2018) or by terming both kinds of republicanism together as “civic republican deliberative democracy” (Peterson, 2011, Ch. 5).

However, representative theorists tend to differentiate their ideas from current republicanism (Sandel, 1996; Sustein, 1988) because of the difficulty of shared identity or values in communities at the present age of value pluralism. Accordingly, the deliberative conception based on liberalism is influential: they frequently mention Rawl’s notion of public reason. His student Joshua Cohen extended the sphere of deliberation to various democratic practices in civil society. Cohen proposed an “ideal deliberative procedure” for public reflection toward the common good under the age of reasonable pluralism, respecting citizens’ autonomy (Cohen, 1989, 1997).

This ideal procedure seems to be inspired by Jürgen Habermas’ idea of ideal speech situation (Habermas, 1996). Inspired by his critical theory, there is a more radical conception of discursive democracy, stressing active citizenship and public discourse as sources of democratic critique and renewal (Dryzek, 1990, 2000).

The most salient difference between republican and liberal or discursive deliberative democracy is that while the former indispensable element is civic virtue, the latter does not necessarily refer to such a substantial ethical conception. Accordingly, while the former embraces the aim of “a comprehensive or thick common good,” the latter holds that of a “non-comprehensive or thin conception of the common good” (Gutmann and Thompson, 2004, pp. 26–27). In sum, although mainstream deliberative democracy shares the public orientation to the common good with republicanism, the former has a weaker ethical orientation than the latter.

## Characteristics of Political Philosophies

### Configuration of Contemporary Political Philosophies

**FIGURE 2:**
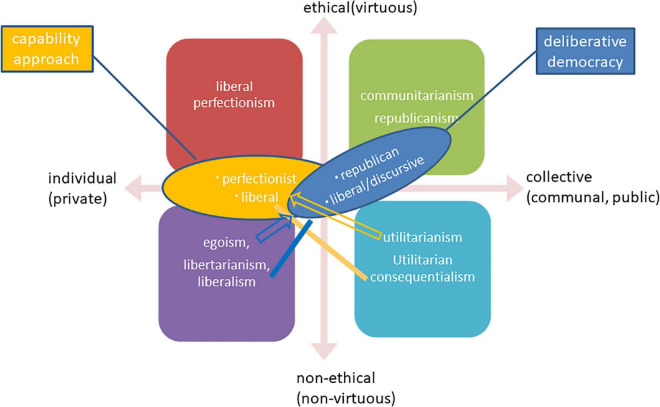
Configuration of political philosophies.

Then, Figure 2 indicates the configuration of contemporary political philosophies by the two axes of “individual (private)/collective (communal or public)” and “ethical (virtuous)/non-ethical (non-virtuous),” found in the debate between liberalism and communitarianism.

First, the major political philosophies mentioned above are configured in the three quadrants. Communitarianism in the first quadrant has two distinguishable features, namely, the ethical (virtuous) and the communal orientation. Within its cardinal concept of the common good, “common” signifies communal orientation, and “good” indicates the virtuous.

In contrast, egoism, libertarianism, and liberalism in the third quadrant are neither communal nor virtuous. These are individualistic. At the same time, egoism and utilitarianism are hedonic; libertarianism and liberalism are non-ethical because they do not assume any particular view of persons.

While utilitarianism or utilitarian consequentialism in the fourth quadrant somehow holds the collective elements in summing the happiness of all people, it lacks the virtuous moment.

Secondly, liberal perfectionism in the section “Liberal Perfectionism” is situated in the second quadrant because this is both individualistic and ethical (virtuous).

Thirdly, the other recent developments in the section “Beyond Deontological Rights-Based Liberalism” can be mapped at intermediate places between plural quadrants. For example, Sen’s capability approach started from utilitarian consequentialism and integrated liberal ideas of rights with consequentialism. It attempts to bridge the consequential fourth quadrant and the liberal third quadrant. Moreover, he introduced some ethical elements such as sympathy, and his approach is related to the second quadrant to some degree. On the other hand, while early Nussbaum’s Aristotelian capability approach is interpreted in the second quadrant as liberal perfectionism, the present approach is mapped in the third quadrant because of its Rawlsian liberal framework.

The deliberative democracy is opposed to aggregative democracy, which is grounded on self-interests, associated with a version of egoism. Accordingly, it holds the collective or the public orientation in the right-hand spheres. Thus, while the republican deliberative democracy is mainly situated in the first quadrant, the liberal version bridges liberalism in the third quadrant with the collective or public domain somewhere between the first and the fourth quadrant.

Thus, it is possible to map these political philosophies in this diagram, indicating the recent noteworthy attempts in relation to the main political philosophies and liberal perfectionism. The most recent developments can be seen as the intermediary or combination of some main typical political philosophies. Moreover, although mainstreams of both the capability approach and deliberative democracy are liberal political philosophies, they embrace another remarkable version tangent to liberal perfectionism or communitarianism: Former Nussbaum’s Aristotelian capability approach is close to the former, and republican deliberative democracy adjoins the latter. This tangency proves that the four quadrants are adequate for mapping these theories.

### Characteristics of the Main Political Philosophies: Citizenship, Justice, and Well-Being

Accordingly, it would be sufficient for this paper to summarize the essential characteristics of main political philosophies from the epistemological or methodological point of view, especially regarding “citizenship, justice, and well-being.”

Table 1, “Basic Characteristics of Main Political Philosophies.” indicates characteristics of these political philosophies, adding (social) conservatism together with the main political philosophies mentioned above. Both libertarianism and contemporary liberalism stemmed from historical liberalism by, for example, J. Locke and J.S. Mill. Contemporary communitarianism derives from classical Greek thought, such as Aristotelian philosophy (Aristotle, 1953/2004). The atomistic worldview is predominant in egoism, libertarianism, and liberalism, while holistic worldview dominates social conservatism. Communitarianism is situated between liberalism and social conservatism, as Etzioni mapped these in his renowned *The New Golden Rule* (Etzioni, 1996). Accordingly, it is sometimes called “liberal communitarianism.”

**TABLE 1:** Basic characteristics of political philosophies.

	Egoism	Utilitarianism	Libertarianism	Liberalism	Communitarianism	(Social) Conservatism
Individualism (atomism)	Strongest	Mild	Stronger	Strong	Mild	Weaker
Self-view	Egoist (selfish)	Selfish	Separable	Separable (abstract)	Relational (encumber ed)	Order-oriented (obedient)
Collectivism (holism)	Weakest	Substantial	Weaker	Weak	Substantial (communal)	Traditional (conventional)
Ethics (morality)	Non	Weak (feeble)	Non or weak (thin)	Non or weak (thin)	Substantial (thick)	Strong (conventional)
Well-being (happiness)	Hedonic	Hedonic	Private	Private	Eudaimonic	Traditional (conventional)
Citizenship	Little concern	Mild	Firm(private)/ weak(public)	Firm(private)/substantial (public)	Substantial (republicanism)	Mild (nationality)
Relationship between citizenship and well-being	Non or weak	Mild (for general well-being)	Enabling possibility	Enabling possibility	Substantial	Weak or mild
Justice	Little concern	Greatest happiness	Liberal (legal rights emphasis on property)	Liberal and distributive (legal rights including welfare rights)	Liberal, distributive, and ethical	Traditional norms, National security and interests
Relationship between the good and justice	Little concern	Yes (exists) (hedonic)	Non	Non	Yes (exists) (eudaimonic)	Yes (exists) (traditional, national)
Relationship between citizenship and Justice	Little concern	Mild	Identical (rights)	Identical (rights)	Substantial	Weak or mild
Relationship between justice and well-being	Non or weak	Firm (direct)	Enabling possibility (indirect)	Enabling possibility (indirect)	Substantial (direct)	Mild (traditional order, national interests)
Relationship between citizenshi/justice and well-being	Non or weak	Mild or firm	Enabling possibility	Enabling possibility	Substantial	Weak or mild

These philosophies can be classified from their human orientations and the corresponding epistemological (or methodological) viewpoint “Individualism (Atomism)/ Collectivism (Holism).” Psychological and ethical egoism is strongly individualistic and hardly communal, and their self-views are “egoist” or “selfish” because they suppose that people act in self-interest or for hedonic pleasure; they are epistemologically based on the atomistic world-views. Since Epicurus in ancient Greek, various theories have more or less associated some forms of egoism: for example, psychoanalysis and behaviorism in psychology, neo-classical economics, and rational-choice theory in political science.

In contrast, utilitarianism is based on the sum of the happiness of individuals, and therefore mildly individualistic and substantially collectivistic (holistic): its self-view is “selfish” in valuing self-interests or pleasure. Its collective aspect is criticized by libertarianism and liberalism. Their self-views are “separable” entities, and the liberal self-view is also “abstract” because, for example, Rawls’ theory assumes the hypothetical situation under the veil of ignorance.

The individualistic and collective orientations of libertarianism and liberalism are respectively “strong” and “weak”. The individualism of libertarianism is more potent than that of liberalism: the collectivism of the former is weaker than the latter because liberalism results in some concern for the welfare of the poor, in contrast to libertarianism. Social conservatism is on the opposite side of liberalism and libertarianism, and the individualistic and collectivistic orientation is respectively “weak” and “strong”. Liberal communitarianism is in the middle ground between liberalism and conservatism. Accordingly, its individualism and collectivism are respectively “mild” and “substantial (considerable)”: its collective orientation signifies the “communal” element as can be seen in various communities, and its self-view is “relational” or “encumbered”. The individualistic and collectivistic orientations of social conservatives are respectively weaker and more potent than those of communitarianism. Its self-view is “order-oriented,” that is, “obedient” to authority, and its solid collective orientation is “traditional” or “conventional”.

The ethical features of these political philosophies are closely related to the Hedonia/Eudaimonia mentioned above. The well-being of egoism is “hedonic” as well as that of utilitarianism, while that of communitarianism is “eudaimonic.” Both libertarianism and liberalism have no particular conception of well-being, but they regard the pursuit of happiness as “private” matters, which should have no relation with public decisions. The conception of well-being held by conservatism is culturally “traditional” or “conventional.” Accordingly, there is almost no ethics or morality in egoism, and they are weak or feeble in utilitarianism because utilitarian morality relies on hedonic pleasure. They are also non in libertarianism and liberalism because they have “no” connection with particular morality, or they are “weak” or “thin” because the conception of rights is sometimes considered thin ethics (Waltzer, 1996). In contrast, communitarian ethics or morality is eudaimonic, and therefore “substantial” and “thick.” On the other hand, socially conservative ethics or morality is also potent but “conventional” rather than philosophical.

### Relationships Among Citizenship, Justice, and Well-Being

The concept of citizenship is “composed of the three main elements or dimensions”: (1) legal citizenship of rights status classically formulated by T. H. Marshall (1950/1992) as ‘‘civil, political, and social,’’ (2) political citizenship as agents actively participating in politics, and (3) ethical or social citizenship as membership in a political community.^3^ This manuscript focuses on the former two, called “legal citizenship” and “political citizenship” hereafter.

The concept of justice also includes these meanings: legal justice, political and economic justice, and ethical justice. Legal justice is closely associated with the concept of rights for some liberty. Political and economic justice has had a connection with distributive justice since the time of Aristotle, and the central theme of justice in politics is still distributive justice in contemporary political philosophy. Ethical or moral justice signifies the quality of being morally fair and right from some ethical or transcendent point of view. So then, these three aspects will be called “liberal justice,” “distributive justice,” and “ethical justice”.

Psychological or ethical egoism has little concern for these because activities related to citizenship or justice require time and energy, which are not part of their interests: egoists usually have no concern with these, and they use legal rights as they need them for themselves. So then, there is typically “no” relationship between citizenship/justice and well-being, or if there is one, it is “weak”.

Utilitarianism does not necessarily emphasize citizenship but sometimes recognizes its value (especially legal citizenship) because its preservation can contribute to people’s general happiness. Accordingly, their commitment to citizenship is “weak” or “mild,” and its relationship to citizenship and well-being is “mild” in the contribution of citizenship to general well-being.

Utilitarianism judges justice by the utilitarian principle, and its relationship between good and justice (the right) exists because the maximization of “hedonic” happiness (the good) is justice. As the good of the people concerned signifies hedonic well-being, its relationship between justice and well-being is also “firm” and “direct”. However, as its commitment to citizenship is “mild”, its relationship between citizenship and justice is also “mild” because citizenship is deemed to contribute to the maximization of happiness only to some degree.

Both libertarianism and liberalism depend on the concept of rights, and they regard legal citizenship and liberal justice as necessary. Nevertheless, libertarianism mainly values economic, legal rights such as property rights, while liberalism also values welfare rights as distributive justice. Accordingly, their commitments to (legal) citizenship are “firm” in private spheres, but that of libertarianism and liberalism are, respectively “weak” and “substantial” in public or social spheres.

Their relationship between citizenship and well-being lies in their “enabling possibility” rather than the existence or the degree such as weak/strong. The reason is that legal citizenship enables citizens to pursue happiness by using their legal rights, but that its existence does not necessarily guarantee the realization of well-being.

Their concepts of justice are almost equivalent to legal rights, and they are not related to the good, and there is no relationship between good and justice, as explained in the section “The Debate Between Liberalism and Communitarianism: Justice and the Good Life”.

From their perspective, citizenship mainly signifies the legal aspect, namely, rights, and justice is largely equal to “legal rights”. Therefore, concerning their relationships between citizenship and justice, former conceptions are identical to the latter. Accordingly, their relationships between justice and well-being are equal to those between citizenship and well-being, namely “enabling possibility”. However, these are “indirect” relationships because rights as justice do not necessarily lead to well-being but only prepare the conditions for an individual’s well-being.

Nevertheless, while libertarianism and some version of liberalism tend to disregard political citizenship, liberal republicanism respects it as the execution of legal and political rights. Accordingly, there can be a mild relationship between justice and citizenship in liberal republicanism, in contrast to libertarianism and non-republican liberalism.

Furthermore, communitarianism evaluates highly political citizenship, accompanied by civic virtues/activities, as well as legal rights. Therefore, its commitment to citizenship is most “substantial”. Moreover, as civic activities are thought to promote people’s well-being, the relationship between citizenship and well-being is also “substantial”.

The conception of justice in communitarianism is “ethical” as well as legal and distributive, and its relationship between good and justice exists (“yes”). The good is based on the philosophical concept and “eudaimonic,” as was explained before. Its relationship to citizenship and justice is different from that found in the relationship in libertarianism and liberalism. Communitarian justice is both legal and ethical, and its citizenship includes not only legal citizenship but also political citizenship. Political active citizenship tends to contribute to realizing justice; the relationship between the two is “substantial”.

Moreover, its relationships between justice and well-being are also “substantial” because its ethical justice contributes to realizing the common good, almost equal to public well-being. Thus, the relationship is “direct” in that justice directly leads to well-being compared to the indirect mode of the two rights-based theories.

The commitment of conservatism to citizenship is “mild” because they tend to disregard fundamental human rights and active citizenship. They instead value duties and the concept of nationality. Accordingly, its relationship between citizenship and well-being is “weak” or “mild” because of its disrespect of citizens’ rights. Its justice depends on the “traditional norms” and national “security/interests”. Accordingly, its relationship between the good and justice “exists (yes)” on the condition that the good is the “traditional and national” in contrast to the communitarian “eudaimonic” conception.

Its relationship between citizenship and justice is “weak” or “mild” because simple nationality is not necessarily related to substantial justice. Its relationship between justice and well-being is “mild” because its justice of traditional norms and national security/interests are respectively associated with its well-being of traditional order and national interests, but only to some extent. The reason is that the national kind of well-being sometimes corresponds to the public well-being of citizens but that the former can contradict the latter in some cases, for example, in which states begin unnecessary wars for the interests of the military-industrial complex in the name of protecting their national interests.

In summary, the relationships between citizenship/justice and well-being in each of the political philosophies are “non or weak (egoism)”, “mild or firm (utilitarianism)”, “enabling possibility (libertarianism and liberalism),” “substantial (communitarianism),” and “weak or mild (conservatism)”. Consequently, the interdependence among citizenship/justice and well-being is the most substantial in communitarianism and the second strongest in utilitarianism. On the other hand, the interdependence in libertarianism and liberalism remains a possibility; conservatism is the second weakest; egoism is the weakest.

Moreover, the relationship between citizenship and justice is mild in utilitarianism and substantial in communitarianism. Therefore, interdependence among citizenship, justice, and well-being is the most substantial in communitarianism.

The reason for the difference related to interdependence can be summarized as follows. Utilitarianism has been historically associated with happiness, while liberalism and libertarianism rely on the concept of rights, which is almost equal to the concept of justice in these philosophies. Accordingly, utilitarianism is a philosophy of well-being, while liberalism and libertarianism are philosophies of justice.

Accordingly, there is little connection between well-being and justice in these philosophies because these are incompatible. Libertarianism and liberalism criticize utilitarianism because the maximization of happiness or utility can infringe on fundamental human rights. On the contrary, the latter criticizes the former because the two deontological philosophies neglect consequential well-being.

In addition, utilitarianism emphasizes (hedonic) well-being but does not emphasize citizenship, while libertarianism and liberalism evaluate legal citizenship but do not take well-being into account. As a result, one is contradictory to the other: the connection between citizenship/justice and well-being is not strong between citizenship and well-being (utilitarianism) or between justice and well-being (libertarianism and liberalism).

In contrast, communitarianism is intrinsically the political philosophy of citizenship most attuned to increasing well-being, and it can connect an idea of justice with well-being. It respects fundamental human rights in its liberal wing and well-being in its communal wing. Therefore, citizenship and justice are not only compatible with consequential well-being but also essential for the latter because they enable the realization of public well-being, namely, the common good. Therefore, citizenship and justice are significant ingredients of civic life and politics. It follows from these arguments that the interdependence among citizenship, justice, and well-being is the most substantial in communitarianism.

## The Relationship Between Political Philosophies and Positive Psychology

### The Ethical and Communal Orientations in Positive Psychology

It will be helpful to offer an inter-disciplinary framework depicting the correspondence between political philosophy and positive psychology to develop the collaboration between the two for pursuing the issues such as citizenship, justice, and well-being. Positive psychology can offer empirical evidence or evidence-based theories corresponding to the two axes of contemporary political philosophies.

First, regarding the first ethical axis, in addition to the eudaimonic well-being mentioned above, Martin Seligman and Christopher Peterson proposed the classification of virtues or character strengths in the name of Value in Action Inventory (VIA). They classified character strengths under six virtues: wisdom and knowledge, courage, love and humanity, justice, temperance, and transcendence (Peterson and Seligman, 2004). These are assumed to be universal through history in the whole globe, and this idea can be regarded as a scientific psychological formulation of virtue ethics. They suppose that human well-being is grounded in them.

Seligman’s theory of well-being (PERMA model) in *Flourish* (Seligman, 2011) is multi-dimensional and contains the elements of *eudaimonia*, especially in meaning (M) and engagement (E). In addition, his close collaborator, a philosopher J. O. Pawelski pushes forward the idea of “eudaimonic turn” in humanities such as literary studies (Pawelski and Moores, 2013). Moreover, other eminent psychological theorists such as Carol Ryff and Richard Ryan and Edward Deci claim that their theories (psychological well-being, self-determination) align with eudaimonic well-being or flourishing in Aristotelian philosophy (Ryan et al., 2013; Ryff, 2013).

The eudaimonic turn in positive psychology is in tune with communitarianism and liberal perfectionism (the upper half in Figure 2): psychological studies can provide these philosophies with scientific corroboration or supporting evidence.

Secondly, positive psychology accumulated extensive evidence that a good human relationship is significant for well-being, regarding the second communal axis. Accordingly, for instance, a positive relationship is one of the pillars, for example, in Seligman’s well-being theory, Ryff’s psychological well-being, and Ryan and Deci’s self-determination theory.

Human relationship in these theories is associated with community or society in the philosophical conception of relationality. As the communitarian view of persons is relational (see section “Relationships Among Citizenship, Justice, and Well-being”), political philosophy can be regarded as a relational public philosophy. Correspondingly, in psychological research, “relational welfare” has already been explored regarding the co-creation of health and well-being for all (Heimburg and Ness, 2020).

Concerning the relational aspect of society, as was touched on cursorily in the section “Criticism Against Positive Psychology and Its Two Frontiers”, an eminent social psychologist Corey Keyes pushed forward a social model of well-being by proposing the measure of “social well-being.” This scale measures the collective orientation for the community, society, and the world (Keyes, 1998, 2005).

While much community psychology intervention such as prevention is still “individualistic or micro-centere micro-centered” (Prilleltensky and Nelson, 2009, pp. 127-129), critical community psychology offers theoretical discussions of community well-being. For example, Isaac Prilleltensky and Ora Prilleltensky pointed to the three primary sites of well-being: personal well-being, organizational well-being, and community well-being (Prilleltensky and Prilleltensky, 2006, p. 11). They accomplished a comparative analysis across countries regarding community well-being and concluded that the more egalitarian countries are, the better health is. According to them, the three key determinants of health and well-being are poverty, power, and participation; critical consciousness about the three determinants, critical experiences, and critical actions are vital to overcoming oppression and exploitation.

In addition, there was also the concept of the common good in the values of organizational well-being and community well-being (Prilleltensky and Prilleltensky, 2006, p.13, Table 1). Accordingly, the conception of community well-being is quite in line with communitarianism. The interdependence of the three types of well-being makes us recognize that private well-being is interwoven with public well-being. Since liberalism and libertarianism separate the two by segregating public decisions from the private understanding of “good life,” they cannot increase public well-being by public decisions based upon some values of personal well-being. In contrast, communitarianism admits various public policies grounded on them. Therefore, it can activate the synergy between personal well-being and public well-being. Moreover, as they emphasized participation and actions, their arguments align with communitarian republicanism.

Furthermore, the idea of positive critical psychology recently emerged. Its handbook (Brown et al., 2018) contains a foreword by Prilleltensky and related articles suggesting a collaboration between community psychology and positive psychology (Martino et al., 2018). The proposed banner of integrating the two approaches, “community positive psychology” or “community-based positive psychology” (Martíniz and Martino, 2018), is almost equal to positive communitarian psychology.

In addition, the pioneering work by Jonathan Haidt (2012) on the relationship between psychology and political philosophy is worth noting. His study focuses on the moral foundation of political philosophies of conservatism, liberalism, and libertarianism. Although this research did not deal with communitarianism, it provides insights into the psychological background of various political philosophies.

These empirical psychological studies can increase or decrease the relative reliability of specific political philosophy in Figure 2. For example, the eudaimonic ethical moment of positive psychology aligns with liberal perfectionism, but it does not necessarily fit its communal moment. On the other hand, the communal developments above have much in common with the communitarian political philosophy. Such a correspondence seems to increase the credibility of some in contrast to different political philosophies.

### Clarification of Political Philosophies and Positive Political Philosophy

This recognition of the association between positive psychology and political philosophies makes the theoretical character of political philosophies clearer from the perspective of the former. The tenet of positive psychology is that it studies the positive side of human mental conditions. In contrast, “psychology as usual” has focused on the negative side of mental disease for its remedy. This characterization is helpful for grasping the opposition between the two rights-based deontological political philosophies (libertarianism and liberalism) and communitarianism.

**FIGURE 3:**
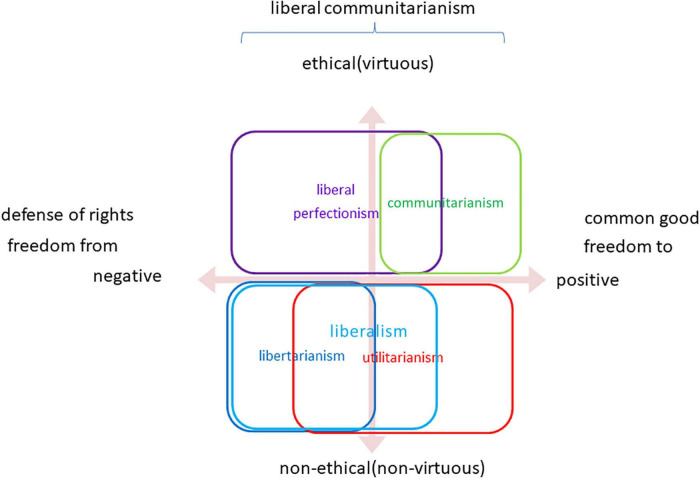
Positive political philosophy.

Figure 3 indicates the configuration of the main political philosophies by using the two axes deriving from the psychological arguments: “negative/positive” and “non-ethical (non-virtuous)/ethical (virtuous)”. The vertical axis is the same as Figure 2. Utilitarianism is hedonic (anti-virtuous), and its goal of maximizing hedonic well-being is principally positive. Accordingly, this is mapped basically in the fourth quadrant. Nevertheless, the formula includes the negative element “sum of happiness-(minus)sum of misery”. Therefore, it is also related to opposing spheres in the third quadrant as “negative utilitarianism.”

Similarly, libertarianism and liberalism are non-ethical and mapped in the lower half, as indicated in Figure 2. Concerning the horizontal axis, the central theme of historical liberalism in Western history is the defense of individual rights from its infringement by the state. The coercive power’s invasion or oppression of liberty is a negative phenomenon. Various autocracies or dictatorships embody the danger. Therefore, liberalism, in the broad sense, is a political philosophy whose main aim is to prevent the appearance of such negative politics.

This element corresponds to “negative liberty” (Berlin, 1969), which is expressed by “freedom from” such as “freedom from evil powers” in the literature of political philosophy. Both libertarianism and liberalism are decedents of historical liberalism, and they share the conception of negative liberty. In particular, while liberalism embraces a limited degree of positive concern because of its justification of welfare policy, libertarianism lacks the positive element.

In contrast, communitarianism is both virtuous and positive and mapped in the first quadrant. The aim of the common good is positive: it indicates various desirable things, such as a suitable environment, peace, and welfare, for people in the community. This element is equal to “positive liberty” (I. Berlin), which is expressed by “freedom to.”

Accordingly, utilitarianism and communitarianism are positive political philosophies. These are teleological because their purposes were maximization of happiness (utilitarianism) or the common good (communitarianism). While the former depends on the hedonic conception of happiness, the latter requires people’s virtues, including civic virtue. Accordingly, these are, respectively mapped in the fourth and the first quadrant: the attention to human nature corresponds to its psychological counterpart, namely, positive psychology.

Positive psychology sometimes encounters criticisms because it neglects the importance of curing mental illness’s negative phenomenon. Likewise, liberalists and libertarians attack utilitarianism and communitarianism as follows: Their pursuit of positive ideals may lead to negative politics such as repression, dictatorship, and violation of human rights.

Positive psychologists reply to the reproach that they do not deny the importance of psychology as usual, and they are only adding a new field of inquiry to it. Seligman and others suggested the goal of “a balanced psychology” as “an integrated, balanced field” (Seligman et al., 2004). Similarly, communitarians refute the liberal and libertarian charges in that they also attach importance to rights and liberty, just as conventional psychology is still vital from positive psychology. For example, Etzioni distinguished their ideas from social conservatives (Etzioni, 1996). While social conservatives surely belittle individual’s autonomy, rights, and liberty, communitarians balance rights with responsibility. The term “liberal communitarianism” signifies an integration of preventing the negative and pursuing the positive. It thus tries to embrace both negative and positive elements in some way. This vision for the integrated, balanced political philosophy is just as balanced psychology.

### Inter-Disciplinary Framework of Political Philosophy and Psychology

On the other hand, such a philosophical typology can inspire psychology. First of all, “psychology as usual” (the third quadrant in Figure 1)is to positive psychology what libertarian and liberal political philosophies (the third quadrant in Figure 2) are to utilitarian and communitarian philosophy (the fourth and first quadrant in Figure 2).

Positive individual psychology (the second quadrant in Figure 1) has already developed with utilitarianism as hedonic psychology (see section “Positive Psychology and Utilitarian Tradition”). However, on the other hand, positive psychology has developed toward perfectionist direction by the conception of eudaimonic well-being. Therefore, this kind of theory is “positive perfectionist psychology” or “individual eudaimonic psychology,” corresponding to liberal perfectionism (the second quadrant in Figure 2).

Positive collective psychology (the first quadrant in Figure 1), including positive political psychology, corresponds to utilitarian and communitarian political philosophy, as was suggested in the last section. While hedonic psychology can develop collectively (the fourth quadrant in Figure 2), positive psychology can develop toward the communal or republican direction (the first quadrant in Figure 2). This vision of “communal eudaimonic psychology” is, as it were, “positive communitarian psychology.” It straightforwardly leads to psychology for the common good.

Then, positive political psychology can develop in two directions. First, it is principally related to positive collective psychology, corresponding to utilitarianism and communitarianism (the right side in Figure 2). Nevertheless, it also has to do with individual psychology because it is associated with libertarianism, liberalism, and liberal perfectionism (the left side in Figure 2). In particular, liberal perfectionism is related to positive individual psychology, which has to do with positive political psychology.

In addition, there may be developments of political psychology inspired by the other theories in Figure 2. In the first place, the capability approach has already impacted well-being studies as the Human Development Index. Furthermore, the psychological dynamics during the deliberation process would be a vital research theme for developing deliberative democracy. Thus, the capability approach or the deliberative democracy can stimulate the progress of positive collective psychology.

Thus, the framework illustrated in the figures and the table makes it possible to summarize the key arguments of this paper. First, as Figure 1 shows, it is desirable to explore positive collective psychology on the basis of the development of positive individual psychology (from individual psychology as usual) and collective psychology as usual, such as social psychology. This new development enables positive psychology to overcome the charge against its character as Western-centered individual psychology (see section “Criticism Against Positive Psychology and Its Two Frontiers”).

Secondly, although positive psychology has been associated with utilitarianism in its early stage, it turned to have links with other political philosophies after the conceptual emergence of eudaimonic well-being (see section “Positive Psychology and Utilitarian Tradition”). Figure 2 indicates contemporary political philosophies, including their recent developments (see section “Major Political Philosophies and Recent Developments”), by the two axes of “individual (private)/collective (communal or public)” and “ethical (virtuous)/non-ethical (non-virtuous).” The four quadrants correspond to communitarianism, liberal perfectionism, egoism/libertarianism/liberalism, and utilitarianism. Capability approach and deliberative democracy are situated somewhere between these four (see section “Configuration of Contemporary Political Philosophies”).

Therefore, it is sufficient to focus on major political philosophies to investigate the relationship between citizenship, justice, and well-being. Then, thirdly, Table 1 summarizes their basic characteristics and the relationship (see section “Characteristics of the Main Political Philosophies: Citizenship, Justice, and Well-being”). As positive psychology embraces the ethical and communal orientation along with the two axes in Figure 2 (see section “The Ethical and Communal Orientations in Positive Psychology”), the framework consisting of the figures and the table will benefit empirical research of positive political psychology.

Fourthly, this framework can also illuminate political philosophies from the perspective of psychology. Figure 3 maps principle political philosophies by two axes of “negative/positive” and “ethical (virtuous)/non-ethical (non-virtuous).” The former horizontal axis corresponds to the “freedom from” and “freedom to,” which are the fundamental concepts of political philosophy: these are parallel to the opposition between libertarianism/liberalism and utilitarianism/communitarianism.

### Multi-Disciplinary Development for Common Good as Collective Well-Being

This clarification and classification of political philosophies can inspire positive psychology or psychology for the common good. For example, empirical research on the relationship between citizenship, justice, and well-being will be possible, bearing the classificatory framework in mind. This kind of empirical research will benefit remarkably political philosophy and, furthermore, social sciences in general.

For example, Harold Lasswell’s pioneer work *Psychopathology and Politics* (Lasswell, 1930/2020) applied clinical psychology concepts to predict and avoid societal and political conflicts. This book is the classical work of political science for preventing negative politics, utilizing psychology as usual.

On the other positive side, while the contents of the common good in philosophical or theoretical discussions in social sciences are abstract in most cases, psychological concepts such as well-being can clarify its effects or levels and make measurement possible. This vision may make the unfulfilled idea of positive social sciences a reality.

For this purpose, it is worthwhile to introduce the concept of collective well-being. This term was proposed for measuring the overall “health” of a community (Roy et al., 2018), and it can be applied to various institutions in society (Waters et al., 2021). This term is close to community well-being mentioned above, and it can be replaced by “common well-being” or “public well-being” in the context of political philosophy and thinking about the common good.

From this perspective, overall well-being or general well-being embraces both individual and collective aspect, and it is affected by “individual well-being” and “collective well-being,” each of which corresponds to positive individual psychology and positive collective psychology. Positive individual psychology verified that individual factors influence individual well-being, illustrated by a happy chart consisting of set points by biological genes, circumstances, and intentional activities (Lyubomirsky, 2007, p.39). The happiness formula expresses this relationship by Seligman (Seligman, 2002, p.45): H(enduring level of happiness) = S(set range) + C(circumstance) + V(voluntary control). Despite the recent critical revision of interpretation or clarification regarding this,^4^ it is still helpful to bear the three factors in mind.

Similarly, positive collective psychology can suppose that collective well-being is affected by culture, society, and politics (or policy). It would be natural to conceive the following correspondence between an individual and a collective factor: biological gene and culture, circumstance and society, and voluntary control and politics (or policy). Then, the collective well-being may be expressed by a formula: CoW(level of collective well-being) = CuW(cultural well-being) + SoW(social well-being) + PoW(political well-being). While cultural well-being is associated with a cultural set range, social well-being depends on social circumstances, and political well-being can change by voluntary collective control of policies.

Accordingly, the introduction of the idea of political well-being may be effective just as community well-being or social well-being. First, citizenship and justice can affect the level of political and social well-being, then collective well-being, and finally, overall well-being. The increase of collective well-being empirically indicates the level of realizing the common good.

In the past, Sen and Nussbaum’s capability approach has influenced not only social sciences but also well-being research. On the other hand, Rawls referred to moral psychology in his *Theory of Justice* (Rawls, 1971, section 69, 75). Habermas introduced and reconstructed Lawrence Kohlberg’s empirical psychological model of moral development for creating discursive ethics in the critical theory (Habermas, 1979).

Likewise, political philosophy can inform psychology, and psychology can invigorate political philosophy through the dynamics of this interdisciplinary framework. As a result, a political philosophy may emerge grounded in the empirical science of psychology, which may develop based on inspiration from political philosophy. In sum, these would be scientific philosophy and philosophical science.

The map of the correspondence between the two disciplines may well prompt new explorations in both fields. Moreover, this collaboration of the two disciplines will also impact social sciences in general. Consequently, this inter-disciplinary framework will accelerate multi-disciplinary development and enable us to proceed toward a psychology for the common good.

## Author Contributions

MK conducted this research with the assistance of Hirotaka Ishikawa (graduate student of Graduate School of Humanities and Studies on Public Affairs, Chiba University).

## Conflict of Interest

The author declares that the research was conducted in the absence of any commercial or financial relationships that could be construed as a potential conflict of interest.

## Publisher’s Note

All claims expressed in this article are solely those of the authors and do not necessarily represent those of their affiliated organizations, or those of the publisher, the editors and the reviewers. Any product that may be evaluated in this article, or claim that may be made by its manufacturer, is not guaranteed or endorsed by the publisher.

## Footnotes

1 The following representative political philosophies are summarized and discussed in Michael Sandel’s much-acclaimed book on justice (Sandel, 2009) for general readers. There are other philosophies such as Marxism, multi-culturalism, feminism, post-modern theories for investigating various issues in the contemporary world (Kymlicka, 2001).

2 This concept was proposed in my presentation “Possibility of Positive Political Psychology” in the 6th World Congress of International Positive Psychology Association, 19th, July 2019.

3 Stanford Encyclopedia of Philosophy, 2006, 2017, https://plato.stanford.edu/entries/citizenship/.

4 Sonja Lyubomirsky, the creator of the happy chart, expressed the modification including the withdrawal of approximate percentage of each factor at the 6th World Congress of International Association of Positive Psychology (July, 2019) in response to criticisms.
